# Action of Quinine

**Published:** 1863-09

**Authors:** J. Hughes Bennett


Action of Quinine.—That quinine is a tonic I have long had reason to doubt. Quantities of this valuable drug appear to me to be annually wasted in administering it in all sorts of affections as a simple means of increasing appetite or communicating strength. I consider it a vegetable alkaloid, which, like others of its class, operates on special parts of the nervous system through the blood vessels. Why it does so we are ignorant. That vegetable poisons do possess this influence constitutes an ultimate fact in the science of therapeutics. But in a similar manner to that in which opium operates on the brain, strychnine and hemlock on the spinal cord, and other drugs operate on other parts of the nervous system, so, I believe, quinine acts on the ganglionic system of nerves, controlling and diminishing those phenomena of fever which physiology has proved to be produced by their irritation or injury.—*(J. Hughes Bennett) Lancet.*

